# Integrated Design of Financial Self-Service Terminal Based on Artificial Intelligence Voice Interaction

**DOI:** 10.3389/fpsyg.2022.850092

**Published:** 2022-03-29

**Authors:** Huizhong Chen, Shu Chen, Jingfeng Zhao

**Affiliations:** ^1^College of Economics and Management, Northwest University, Xi’an, China; ^2^College of Accounting, Zhanjiang University of Science and Technology, Zhanjiang, China

**Keywords:** artificial intelligence, intelligent voice, financial self-help, integrated design, terminal

## Abstract

Integrated design of financial self-service terminal based on artificial intelligence voice interaction with the rapid development of science and technology, artificial intelligence technology is deepening in the field of intelligence and automation. The financial industry is the lifeblood of a country’s economy, with great growth potential and high growth rate. The integrated design of intelligent financial self-service terminal has become an important topic in the field of rapid development of social economy and science and technology. Therefore, this paper designs the integration of financial self-service terminal based on artificial intelligence voice interaction. First, this paper introduces the meaning and composition of financial self-service terminal integration, then studies the voice interaction principle based on artificial intelligence technology, and designs the integrated structure of financial self-service terminal with voice interaction. After that, this paper makes a series of tests on voice interaction technology, user experience, and the performance of financial self-service terminal. Finally, the test results of voice interaction are as follows: the delay estimation results of voice interaction of the terminal are relatively accurate, and the error points are basically within five sampling points, which indicate that the delay estimation algorithm is practical. The endpoint detection method based on CO complexity can effectively overcome the impact of noise environment on speech endpoint detection system and is suitable for the requirements of robust speech recognition system. Considering that the actual application scenario of voice positioning can judge the speaker’s position and turn to the speaker’s direction during human–computer interaction, the azimuth error is acceptable within a few degrees to meet the application requirements. The direction angle error is acceptable within a few degrees to meet the application requirements. The accuracy of the improved algorithm is improved in intercepting effective speech signals. The terminal has short running time and delay time, small memory, and central processing unit (CPU) occupation and can meet the needs of users. The speech recognition accuracy of the financial self-service terminal basically reaches more than 80%, which can basically meet the daily needs.

## Introduction

The development of artificial intelligence technology is the inevitable result of the progress of human scientific and technological civilization and the improvement in social productivity. With the rapid rise, popularization, and application of science and technology such as computer and network communication, intelligence has become one of the important parts of future production and life, and automatic product design is more and more used in all walks of life (Wei and Kehtarnavaz, 2018). These fields also include a large number of AI robots with high-quality talents, which are in great demand but lack of relevant experience, knowledge, and complex and changeable working environment. Therefore, its development urgently needs research and development.

Many scholars have done relevant research on artificial intelligence speech interaction. As. I et al. provide a method of image-based machine learning. Based on the traditional position orientation system (POS) imaging system, combined with advanced theories such as computer vision and pattern recognition, this technology obtains a new sequence after iterative transformation of the input image and realizes the combination of camera calibration and bug 3D modeling to build the corresponding relationship model between camera and model parameters. Two algorithms are used to calculate the contrast ratio of different light intensity values in the output spectral data and compare the change trend of coherence difference between them (As et al., 2018). M Zhao believes that with the application of new technologies and new algorithms represented by artificial intelligence in scientific research, life, and other fields, people have focused on this emerging research direction and made some progress. Among them, deep learning is a very promising topic worthy of in-depth discussion. A new intelligent robot is formed based on the combination of neural network and fuzzy logic. This new type of intelligent robot is composed of realizing the working mode by simulating the human brain nervous system in the traditional industry, so as to achieve the ability to automatically adapt to the environment. It has the characteristics of high adaptability, high flexibility, and good robustness. Because of its unique advantages, people can quickly perceive the surrounding things (Shan et al., 2019).

Erying Guo invented an artificial intelligence English cross language video translation system using fuzzy computing. Based on MATLAB software, a complete and accurate semantic expression framework is constructed using fuzzy theory and reasoning analysis method. The framework will use the vocabulary, syntactic structure, and inter sentence word order of words as inputs for online learning and calculate the required output information and results. At the same time, combined with the scene characteristics, it will realize the corresponding feedback values in different contexts, so as to provide users with more accurate, practical, and efficient translation services (Guo, 2021). Xu Kun takes machine learning algorithm as the technical cornerstone, integrates artificial intelligence technology and internet of things technology, and studies the advantages of machine learning algorithm in practical application. First, the methods of modeling and design based on feature model classification and cluster analysis are introduced. The data set is trained and calculated by using naive Bayesian network, which is output to the objective function as the prediction result to realize the prediction output, and the input vector is represented by the improved neural network diagram. Finally, the machine learning algorithm framework is established by using fuzzy NOTEL (Kun et al., 2021). P Radanliev proposed a new mathematical method to realize intelligent fuzzy reasoning based on the combination of gray correlation analysis method (k-d lattice method) and artificial neural network algorithm. In the process, two mathematical models, nearest neighbor search technology and genetic algorithm (LIBS), are used to model and judge whether the result is the optimal solution, and the corresponding conclusions and solutions are given (Radanliev et al., 2020). Y Qin proposes an improved TrAdaBoost algorithm based on transfer learning theory. This paper mainly studies the learning rules and methods based on TRADA reference, analyzes the common n-class interpolation algorithms, and compares their advantages and disadvantages. In addition, a new class of multiinput, single output system is established based on spatial arbitrary programming and the combination of n-dimensional description (K-r) and other models. This learning strategy can effectively solve the traditional online line matching problem. By multiplying the linear distance and the weighted average error, the shortest path within the selection range of Timber Research and Development Association (TRADA) is obtained by KXI function (Qin et al., 2019).

Z Hu was used to synthesize poly(3,4-ethylene two oxy thiophene) in situ on the polyurethane foam skeleton, and then, polyvinyl chloride epoxy resin was synthesized with polyethylene as a raw material. Then, different catalysts and reaction conditions were used to carry out experiments. Propylene carbonate was added to the epoxidation polymerization system with propylene carbonate as initiator and dimethyl ether as main solvent. After the preparation of polymer plastics, its properties were studied, analyzed, and discussed; Finally, by changing the variable factors to explore the best process parameters and optimize the formulas of each group, the optimal combination formula is obtained: (synthetic polyvinyl chloride = 5%, polycondensation time 80 min), and the final resin body fraction meets the requirements (Hu et al., 2021). BA Erol studies a new emotion-based collaborative human resource information system (HRIS) perception architecture. First, the ion migration behavior and pscs-cage selective detection model are introduced, then the self-contained psmems environment of pedfd is established, and the CPFS perceptual quality evaluation index system in line with the actual application scenario is constructed based on emotional attitude. Finally, the bidirectional (BDO) algorithm in SAASIM software is used to design the evaluation method based on research object “renewable resources” and scpol technology (Erol et al., 2019).

Al Guzman’s research on source localization mainly introduces several source localization methods, experimental data acquisition and result processing methods, and simulation model research. By comparing different measurement methods, high-precision signal reception is realized and fed back to personal computer (PC), and then, the information of PC is used to finally complete the target localization of the whole system (Verde et al., 2019). Internet of things technology mainly carries out information exchange and communication through sensors, radio frequency identification (RFID), and so on, so as to achieve the purpose of smart home. In recent years, with the combination of wireless sensor networks and mobile internet, various applications based on the internet of things are constantly innovating and rapidly popularizing. At the same time, due to the improvement of people’s awareness of security and the increasing demand for their own property protection, a new generation of products came into being–new functions such as bluetooth technology (pandomet) and infrared ranging are widely used–which provides a good foundation and promotion for the research of the internet of things. In this regard, to limit the possibility of security attacks or privacy violations, l Verde proposes a mobile voice detection method based on lvir (likanintics), which is composed of hardware circuit and software program. First, a signal acquisition module, micro controller unit (MCU) control part, and display driving system are designed. Second, the data set is discretized and quantified to obtain the interrupt function, and the corresponding eigenvalues are calculated according to this parameter. Then, the digital time-shifting algorithm based on lvir (likanitics) is written on MATLAB platform to realize the mobile speech detection experiment (Guzman, 2019).

The above research on artificial intelligence voice interaction methods has been carried out, and researchers have put forward a lot of relevant work in recent years. However, the existing research work does not consider the impact of artificial intelligence voice interaction at the same time in the integration method of financial self-help terminals, that is, the impact of artificial intelligence voice interaction methods on the integration of financial self-help terminals. In the use of financial self-service terminal services, users are not familiar with other functions, which results in low quality of user experience and affects the customer’s experience. It also includes some errors caused by sound and noise interference, such as reduced fidelity, inaccurate data transmission, and high bit error rate. In addition, there are many problems, such as: the accuracy of speech signal recognition is not high, the understanding of the machine is not deep, and so on.

The innovation of this paper is mainly reflected in the following three aspects: (1) the artificial intelligence voice interaction system is studied. Through the investigation of the integrated design status of intelligent financial self-service terminals at home and abroad, the shortcomings under the current mainstream trend are summarized. (2) This paper proposes an intelligent gesture recognition technology characterized by people-oriented, humanization, and personalization. This method can meet the needs of customers by providing different operation functions on different interfaces. (3) The integration of artificial intelligence technology and financial self-service terminal is combined to realize intelligent financial automatic service through voice communication, so that it can play a great role in daily life and work.

## Discussion on the Integration of Financial Self-Service Terminals Based on Artificial Intelligence Voice Interaction

### Content

The main research content of this paper is artificial intelligence voice communication technology. Based on this, this paper first introduces the relevant basic concepts and development history. Then, through the analysis of the advantages and disadvantages of intelligent terminals at home and abroad, this paper summarizes the existing problems and future trends of domestic intelligent financial self-service tools. When the human–computer interaction is realized by combining the functions of human visual system and auditory perception system, the embedded keyboard and keys are designed, the fingerprint recognition algorithm is used to identify the user’s identity information, the ultrasonic ranging technology is used to detect the vehicle position, and the measured data are transmitted to the machine language processing platform to complete the voice prompt and communication process of a simulated scene.

### Integration of Financial Self-Service Terminals

#### Definition

Financial self-service terminal is an intelligent digital electronic device that applies computer technology to traditional financial institutions, provides remote services and business processing functions, and realizes data exchange or fund settlement between banks, securities, and other institutions. It has voice recognition, data processing, and storage functions and can carry out various business operations. Financial self-service terminal is the core of intelligent financial engineering. It includes voice recognition, data acquisition system, and other functions. It can automatically process all kinds of complex data and store, obtain, and analyze a large amount of original information. When using the software, all tasks can be completed by voice input or playing voice commands. With the development and application fields of artificial intelligence technology and the improvement of people’s living standards, the demand for high-tech products has increased, which not only promotes the rapid rise of “financial electronics” industry, but also provides a good platform for intelligent financial services, so that it can meet the individual requirements and needs expected by various users in daily life (Shan et al., 2019; Jain et al., 2021). The so-called integration, as the name suggests, is to decompose a complex system into functional modules, control modules, and interface units. In this way, work efficiency can be effectively improved. Intelligent voice terminal means that users need to interact with the upper computer of the host when using voice recognition chip.

#### Composition

**FIGURE 1:**
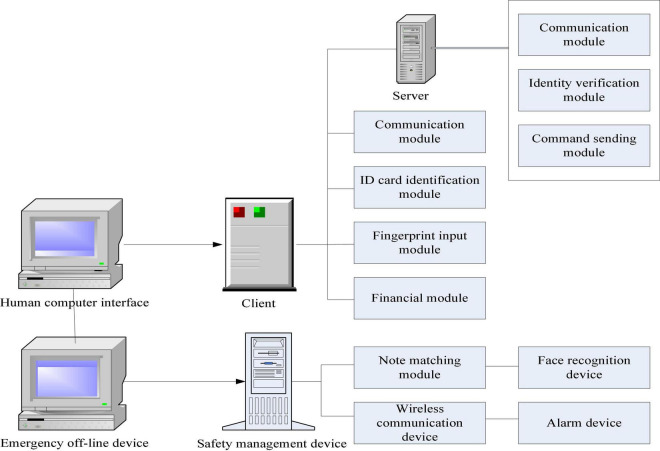
Integration framework of financial self-service terminals.

The system consists of speech recognition module, data processing and analysis module, and interactive interface (as shown in Figure 1). The input part provides users with corresponding intelligent terminal devices, such as fingerprints, palmprints, etc. when biometrics are collected, they are stored as the next functional unit. At the same time, the obtained information can also be read from the database through voice channel, which is extracted and integrated to facilitate subsequent operations. When users want to query or retrieve certain information for a specific target, you need to get the environment information, activity range, and other related attributes of the object. It analyzes the collected information through the corresponding software and then transmits it to the processor to control relevant operations. At the same time, it can also directly read and write or digitally compress the storage space to realize the automatic identification of data and save, process, and print the file content required by users, so as to achieve the purpose of user self-service (Ashwin, 2017; Shirui et al., 2020).

### Artificial Intelligence Voice Interaction

#### Voice Pre-processing

The integration of financial self-service terminals based on voice interaction needs the support of artificial intelligence technology. Next, this paper studies the artificial intelligence technology accordingly. Generally, for feature analysis, good features are the inevitable pre-requisite to achieve the prediction results, and speech signal processing is no exception. In the pre-processing stage, the signal features are generally required to be processed as follows: pre-emphasis, frame windowing, noise reduction, and so on. In the process of digital signal processing, the components of voice information in the high-frequency part are reduced due to the influence of glottic excitation (Miller, 2019; Geibelstefan, 2021). The attenuation of speech signal is the phenomenon of low frequency, large delay, and instability when the sound intensity exceeds the power emitted by the speech signal under a specific condition, or below this threshold. This sound is easy to be ignored and produces noise pollution (Zhang et al., 2017; Dartmann et al., 2019; Ko et al., 2021). Therefore, the first-order digital filter is usually used to adjust the weight of signal processing to improve the characteristics of voice information in the high-frequency part. This paper studies AI speech interaction techniques using descriptive analysis methods. The mathematical definition of first-order digital filter is shown in Equation (1):


(1) $$H\,\left(Z\right)=1-\mu \,z^{-1}$$

When the first-order filter is used, the information of each frame is pre-weighted to reduce the impact on the low-frequency information. Then, the voice information is divided into frames. To make a smooth transition between frames and ensure the coherence of information, overlapping segmentation is usually used, which is called the frame moving speed at the alternation of the front and rear frames. Due to the short-term stability of voice communication, people usually set the frame length at about 10–30 ms, and the frameshift is generally 1.5 of the original frame. Finally, the windowed speech information is obtained by weighting the appropriate window function w (*n*) and each frame signal s (*n*):


(2) $$Z_{\text{n}}=\frac{1}{2}\,\sum_{m=1}^{N-1}\left|s\,h\,g\,\left[x\,\left(b\right)\right]-s\,h\,g\,\left[x\,\left(b-1\right)\right]\right|$$

$$Z_{\text{n}}=\frac{1}{2}\,\sum_{m=1}^{N-1}\left|s\,h\,g\,\left[x\,\left(b\right)\right]-s\,h\,g\,\left[x\,\left(b-1\right)\right]\right|$$

For short-term signal analysis, the selection of window function will have different effects.

#### Feature Extraction

Feature extraction is an important part of the interaction between smartphone certificate-making system and artificial intelligence financial technology. It can analyze the data through the computer and transform it into text, image, or other information forms with a specific meaning. Endpoint checking is a technical means to determine the beginning and end of the voice segment from the voice information, so as to distinguish the silent segment from the audio segment. The voice endpoint checking algorithm mainly checks the endpoint through the simultaneous frequency domain characteristics and statistical characteristics of the conversation voice. Common endpoint detection algorithms include endpoint detection technology based on short-time energy and short-time average zero-crossing rate, endpoint detection technology based on spectral entropy, endpoint detection technology based on complexity, and so on. The so-called complexity means that it is difficult to collect sound, image, or other information due to the change of voice signal in different environments. Speech recognition technology is to extract, classify, and analyze a variety of features. As is known to all, speech is not short-term stable information, so people often use short-term analysis methods when analyzing speech-related features. In other words, assuming that speech is short-term stable, generally speaking, the characteristics of speech short-term analysis include short-term zero-crossing rate, short-term energy, cepstrum distance, etc. (Kurahashi et al., 2017; Jain et al., 2022).

(1) Endpoint detection method based on short-time energy and short-time average zero-crossing rate

For each frame of voice information, the number of signal symbol changes at each acquisition point is the short-term average zero-crossing rate, which also reflects the characteristics of rate change. Obviously, the signal has a higher zero-crossing rate at high frequency, but its zero-crossing rate will decrease at low frequency, so it can be used to roughly infer the spectral characteristics. For discrete signals such as X (*n*), the voiced segment and voiced segment can be distinguished using the short-time energy difference. However, considering that the energy value of voiced segment is much larger than that of voiced segment, the change time of voiced speech and voiced speech can be roughly determined by energy function. If the frame length is *n*, en is used to represent the short-time energy of the speech signal xn (*m*) of the nth frame, and the calculation formula is as follows:


(3) $$Z_{\text{n}}=\frac{1}{2}\,\sum_{m=1}^{N-1}\left|s\,h\,g\,\left[x\,\left(b\right)\right]-s\,h\,g\,\left[x\,\left(b-1\right)\right]\right|$$

The effective length of the signal is n, and the definition of the symbol function is shown in (4):


(4) $$\text{shg}\,\left[\text{x}\,\left(\text{n}\right)\right]=\left\{ \begin{array}{c} 1,x\,\left(n\right)\geq 0\\ -1,x\,\left(n\right)<0 \end{array}\right.$$

The short-term average energy can track the change trend of speech signal, and the difference between voiced frame and unvoiced frame is obvious. Therefore, this feature can be used to effectively identify the effective starting segment of speech. Short time energy is defined as follows:


(5) $$E_{\text{n}}=\sum_{b=n-N+1}^{n}{\left[x\,\left(b\right)\,w\,\left(n-b\right)\right]}^{2}$$

Where, w (n) is the window function.

(2) Endpoint detection method based on spectral entropy

Compared with the background noise, the amplitude dynamic range of voice information is relatively large, so it is considered that the random events of voice information in the voiced stage are relatively large, that is, the entropy is large, whereas the amplitude range is small and the event distribution is relatively concentrated in the voiceless stage, so the entropy is small. The spectral entropy measurement method makes statistics from the frequency domain in the voice channel and then realizes the measurement of dialogue voice endpoints from the spectral distribution probability (Hildebrandt, 2018; Moore, 2019).

The calculation method of spectral entropy is as follows: first, the spectrum s (*k*) of each frame signal s (*n*) is obtained by velocity Fourier transform (FFT), and S (*k*) represents the energy distribution of the frame information at frequency point K. The percentage of various frequency components in the total energy is counted and regarded as the switchboard rate at which all signal energy is concentrated at a certain frequency point. Its probability is defined as follows:


(6) $$P_{\text{i}}=\frac{S\,\left(\text{i}\right)}{\sum \begin{array}{c} N-1\\ k=0 \end{array}\,S\,\left(K\right)}$$

where n is the number of FFT transform points.

Since most of the energy of speech signal is concentrated between 200 and 3,500 Hz, to centrally calculate the spectral entropy to increase the discrimination between speech and non-speech in the probability density function.

The spectral entropy of each frame is defined as follows:


(7) $$H=-\sum \begin{array}{c} N-1\\ K=0 \end{array}\,P_{K}\cdot \text{log}\,p_{k}$$

H represents the meaning of noise and P represents the peak value of sound. The spectral entropy of each frame speech signal is calculated by entropy function, and the starting point of speech is detected by comparing with the threshold.

#### Speech Recognition Technology

Speech recognition is the process of converting audio that contains speech into corresponding text output. Speech recognition technology uses different features of language to process information, which includes text, sound, and image, so as to convert these data into recognizable digital signals. The development of speech recognition technology has witnessed the recognition of a small number of words, specific characters, and single words, which has evolved into the ability to recognize extended words, continuous language, and non-specific language. Every innovation of technical means and algorithms has brought a major breakthrough in the field of human speech recognition. The use of the probabilistic data model is the leading idea of current speech recognition technology. Using the Bayesian principle, speech recognition technology can be expressed as a prediction problem and output text according to the observation sequence. The description of the mathematical formula includes as follows:


(8) $$\overset{⌢}{W}=\underset{W}{\arg\max}\left\{p\left(W|X\right)\right\}$$

X represents the input sequence of observations. W said possible word sequences, P | x (W) is under a given observation sequence x W after probability output sequences of words. Because the p | x (w) is difficult to calculate directly, so using the Bayesian formula, P | x (w) in a given sequence of words, the acoustic model can get the output probability of observation sequence. The probability of P (W) said leave word sequence, only related to the system of language model. 0(x) is a constant term, which is independent of word order (Bagheri and Cheung, 2018; Wilson, 2018).

Speech signal is a kind of change, is influenced by many factors, and has a different state. For example, when people catch a cold, change their pronunciation, or they speak speed is different, so the same pronunciation can have a different status. Gaussian mixture model has a strong ability of fitting which can be adapted to almost any distribution data. By fitting various factors affecting language with multivariate Gauss, and then superimposing all multivariate Gauss, the distribution patterns of language features in different states are obtained. For a Gaussian model with dimension D and component M, it is defined as follows:


(9) $$p\,\left(x\right)=\sum_{b=1}^{\text{b}}\frac{c_{b}}{\left|\sum M\right|}$$

Among μ represents the mean vector and M represents the covariance matrix. The parameters of Gaussian mixture model are mainly estimated by EM algorithm, and the formula is as follows:


(10) $$C_{m}^{\left(j+1\right)}=\frac{1}{N}\,\sum_{I=1}^{N}h_{m}^{\left(j\right)}\,\left(t\right)$$

(11) $${\text{u}}_{m}^{\left(j+1\right)}=\frac{\sum_{i=1}^{N}h_{m}^{\left(i\right)}\,\left(t\right)\,x^{\left(t\right)}}{\sum_{i=1}^{N}h_{m}^{\left(i\right)}\,\left(t\right)}$$

After the acoustic model is determined, the decoding module decodes the current speech signal in combination with the pronunciation dictionary to obtain the corresponding possible word sequence.


$$\sum_{\text{m}}^{\left(\text{j}+1\right)}=\frac{\sum_{i=1}^{N}h_{m}^{\left(i\right)}\,\left(t\right)\,\left(x^{\left(t\right)}-\mu_{m}^{\left(j\right)}\right)\,{\left(x^{\left(t\right)}-\mu_{m}^{\left(j\right)}\right)}^{T}}{\sum_{i=1}^{N}h_{m}^{\left(i\right)}\,\left(t\right)}$$

$$\sum_{\text{m}}^{\left(\text{j}+1\right)}$$

$$=\frac{\sum_{i=1}^{N}h_{m}^{\left(i\right)}\,\left(t\right)\,\left(x^{\left(t\right)}-\mu_{m}^{\left(j\right)}\right)\,{\left(x^{\left(t\right)}-\mu_{m}^{\left(j\right)}\right)}^{T}}{\sum_{i=1}^{N}h_{m}^{\left(i\right)}\,\left(t\right)}$$

(12) $$\sum_{\text{m}}^{\left(\text{j}+1\right)}=\frac{\sum_{i=1}^{N}h_{m}^{\left(i\right)}\,\left(t\right)\,\left(x^{\left(t\right)}-\mu_{m}^{\left(j\right)}\right)\,{\left(x^{\left(t\right)}-\mu_{m}^{\left(j\right)}\right)}^{T}}{\sum_{i=1}^{N}h_{m}^{\left(i\right)}\,\left(t\right)}$$

$$\sum_{\text{m}}^{\left(\text{j}+1\right)}$$

$$=\frac{\sum_{i=1}^{N}h_{m}^{\left(i\right)}\,\left(t\right)\,\left(x^{\left(t\right)}-\mu_{m}^{\left(j\right)}\right)\,{\left(x^{\left(t\right)}-\mu_{m}^{\left(j\right)}\right)}^{T}}{\sum_{i=1}^{N}h_{m}^{\left(i\right)}\,\left(t\right)}$$

H (T) for the current Gaussian mixture distribution hypothesis, j for the current iteration index, and X (T) for time T feature vector. For continuous speech recognition, word order alone cannot solve the problem. In response to this request, a language model is provided (Tribbey, 2017; Shevis, 2018). Given a learning corpus, the role of language models is to calculate the collocation probabilities and parts of speech of words and then predict the most likely combinations of all words based on the predictions of acoustic models and pronunciation dictionaries. Therefore, if the prior knowledge related to a particular domain or task can be calculated, then the language model for solving the most likely phrase problem is the probability estimation problem.


(13) $$P\,\left(S\right)=P\,\left({\text{w}}_{1},w_{2},\ldots ,w_{n}\right)$$

Obviously, Equation (13) there are two disadvantages: (1) parameter space is too big: conditional probability combination is overmuch, amount of calculation is difficult to predict. (2) Data sparse severity: calculate probability may be 0. So as to solve the problem of too large parameter space, Markov hypothesis is introduced, that is, the probability of the current word is only related to the first N-1 words in history and has nothing to do with the first N words and the following words. It is called the NGram model. After the formal model, there is:


(14) $$P\,\left(S\right)=\prod_{i=1}^{\text{n}}P\,\left(w_{i}|w_{i-n+1},\ldots ,w_{i-1}\right)$$

when *n* = 2, it is called bigram model, which can be obtained according to the above formula:


(15) $$P\,\left(w_{1},w_{2},\ldots ,w_{n}\right)=\prod_{i=1}^{n}P\,\left(w_{i}|w_{i=1}\right)$$

Similarly, when *n* = 3, the trigram model includes:


(16) $$P\,\left(w_{1},w_{2},\ldots ,w_{n}\right)=\prod_{i=1}^{n}P\,\left(w_{i-2},w_{i-1}\right)$$

#### Linear Prediction Technology

The basic idea of linear prediction analysis is to use the correlation between speech samples, based on the past sample values, and then predict the current and future sample values. That is, taking the minimum prediction residual as the standard, the linear prediction coefficient is calculated. The characteristics of language information can also be described by prediction coefficients. In speech information technologies such as speech recognition and language fusion, prediction coefficients can be used as language information characteristic parameters for signal processing. P sample values are used to estimate the current or future sample values:


(17) $$\hat{s}\,\left(n\right)=\sum_{i=1}^{p}a_{i}\,s\,\left(n-i\right)$$

where p is the prediction order and AI is the linear prediction coefficient. The prediction residual e (n) is as follows:


(18) $$\varepsilon \,\left(n\right)=s\,\left(n\right)-\hat{\text{s}}\,\left(n\right)=s\,\left(n\right)-\sum_{\text{i}=1}^{\text{p}}{\text{a}}_{\text{i}}\,s\,\left(n-i\right)$$

The transfer function a (z) between the output residual function E (n) and the input function s (n) is defined as follows:


(19) $$A\,\left(z\right)=1-\sum_{i=1}^{p}a_{i}\,z^{-i}$$

To minimize the square value of prediction residual:


(20) $$\varepsilon =E\,\left[\varepsilon^{2}\,\left(n\right)\right]$$

(21) $${\partial \left[\varepsilon^{2}\,\left(n\right)\right]}_{\partial a_{i}}=0,\left(1\leq i\leq p\right)$$

P is the prediction order, which is exactly the number of poles. AI represents the pitch information of speech signal based on the characteristics of speech channel model.

## Experimental Analysis of Financial Self-Service Terminal Integration Based on Artificial Intelligence Voice Interaction

### Voice Interaction Module

**FIGURE 2:**
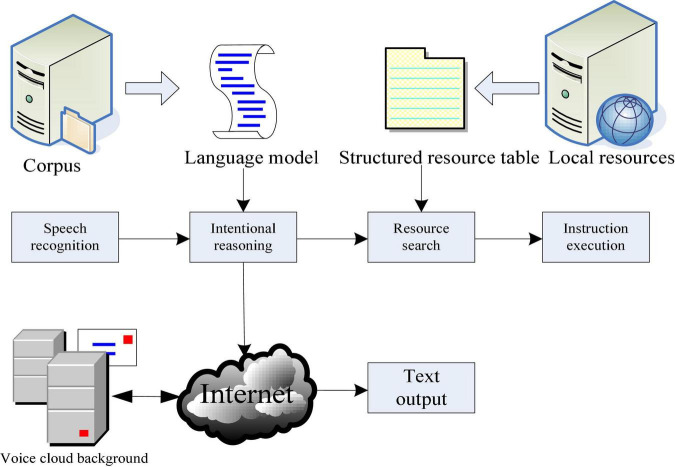
Voice interaction module.

Voice interaction function is the core of this design. It consists of two modules. One is the information exchange between users and others. The other is language signals. The voice communication system mainly includes three parts: language acquisition, data processing, and image recognition. At this point, digital filtering technology and filter principle need to be analyzed and compared to eliminate or reduce noise interference. The last link is data storage and storage for feedback or decision-making in the next calculation, that is, the process of semantic understanding. As shown in Figure 2, after the system obtains the speech recognition results, the next step is to use the local relatively rough language model to infer the speaker’s intention in advance. If the local inference engine accepts the reasoning, it will schedule the local resources through the resource search module to make corresponding decisions. If the speaker’s intention cannot be resolved locally, turn to the cloud background to access the voice cloud service to realize intelligent chat.

### Speech Recognition Technology Analysis

Speech signal is transformed from sound, so we can call it “listening,” and signal-to-noise is called information. In a noisy environment, the speaker and unpleasant and low-quality words will affect the emotion needed in people’s communication. The initial delay of speech signal refers to the collected speech information needed to complete a complete cycle task, which includes the number of corresponding pixels in the image, time range, etc. After each cycle, judge whether to play a video data according to the frame length and threshold. In this paper, the performance of the designed algorithm is tested in indoor and outdoor environments. The main noise is daily noise such as outdoor chassis operation noise, external noisy human voice, indoor mouse click sound, echo, and so on. A total of five groups of experiments were conducted, and each group was conducted three times. The human voice was used as the sound source, and the horizontal distance between the human and the microphone array was 100 cm. Table 1 shows the five-time delay estimation results, which are represented by the number of samples. It can be seen from the table that the time delay estimation results are relatively accurate, and the error points are basically within five sampling points, which indicates that the time delay estimation algorithm is practical.

**TABLE 1:** Time delay forecast results.

Acoustic source	Theory 1	Reality 1	Theory 2	Reality 2	Theory 3	Reality 3
P1	27.5	27.1	34.25	34.54	34.2	35.6
P2	21.3	23.1	23.5	24.2	31.5	33.1
P3	25.5	26.4	54.2	57.6	32.4	32.5
P4	24.1	24.9	53.1	54.5	42.7	44.3
P5	25.3	25.8	60.25	61.4	40.2	43.2

**FIGURE 3:**
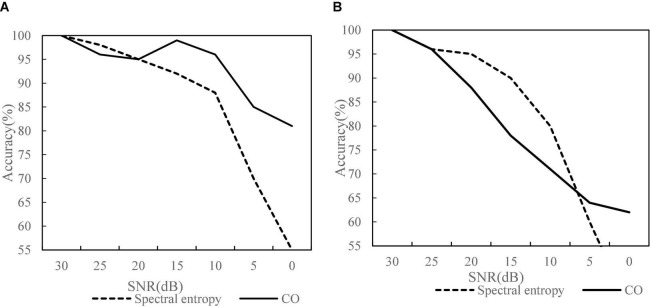
Comparison of detection effects in different environments of speech endpoints. **(A)** Indoor environment. **(B)** Outdoor environment.

The method of endpoint detection based on spectral entropy and cocomplexity is applicable to endpoint detection in the environment with low signal-to-noise ratio, but when the signal-to-noise ratio drops to a certain extent, it can be seen from Figure 3. Under the conditions of stable indoor environment (Figure 3A) and outdoor noise in unstable noise (Figure 3B), the detection results of CO complexity are better than spectral entropy and have high accuracy. Therefore, the endpoint detection method based on CO complexity can effectively overcome the impact of noise environment on speech endpoint detection system and is suitable for the requirements of robust speech recognition system.

Voice signal positioning refers to the use of computer man–machine interface technology to transmit and interact information between the machine and the external environment. The speech recognition system outputs the collected information to the processor for processing. In the process of speech signal strength measurement, we use parameters such as short distance, long time width, and high-frequency band to calculate the corresponding threshold. Because this design adopts the endpoint detection and analysis method to judge whether there is sound, no noise interference, sound generation, and so on, at the same time, considering the influence of noise and other factors, we decide how to select appropriate parameters to determine a correct signal, so as to achieve the purpose of the sound source location. It can be seen from Table 2 of the experimental results that the azimuth and pitch errors are relatively small. Considering that the actual application scenario of voice positioning can judge the speaker’s position and turn to the speaker’s direction during human–computer interaction, the azimuth error is acceptable in a few degrees to meet the application requirements.

**TABLE 2:** Sound source positioning data.

Acoustic source	The theoretical orientation angle	The actual orientation angle	Theoretical elevation angle	Actual elevation angle
P1	4.23	2.14	62	65.24
P2	14.21	11.3	45.24	50.14
P3	23.10	23.54	24.35	21.42
P4	−30.25	−14.25	31.54	34.24
P5	−4.36	−6.25	58.67	61.41

**FIGURE 4:**
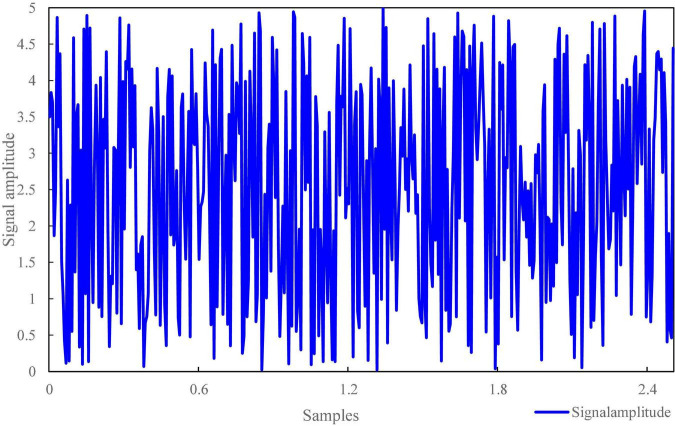
Fluctuation of voice signal.

**FIGURE 5:**
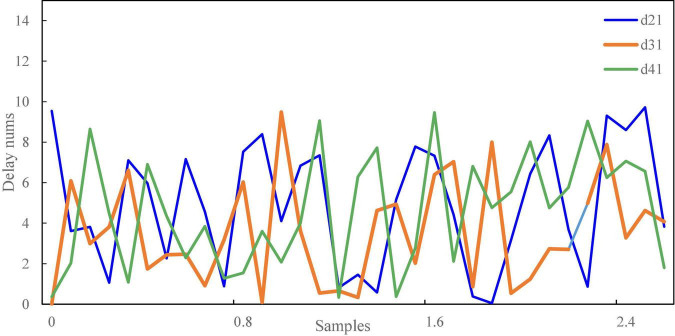
Speech signal delay results.

The signal-to-noise ratio of voice signal is an important factor that users first consider when using intelligent terminals for self-service financial transactions. In terms of speech recognition, it can be seen from the experimental results in Figures 4, 5 that the used audio signal and low-frequency noise have certain common effects. The speech signal fluctuations in Figure 4 range from 0–5 Hz, whereas the speech signal delay time in Figure 5 is from 0–10 ms. What it perceives to us is that there is no quiet or irregular motion state information at a time point (such as when talking and listening), which has the distribution characteristics of random factors to a great extent, followed by coherent noise interference. Speech contains a large number of coherent noise signals and aliasing noise signals, which will make that the image to be tested has a certain common mode relationship.

The key of this design is the implementation of speech recognition algorithm. After a large number of experiments, it is found that the accuracy of speech recognition also affects the integration process of financial self-service terminals to a great extent. In the speech interception experiment, this paper first considers from two aspects. The first is to analyze and judge the information input by the user. The second is to extract the key features from the data and integrate them. The original data to be intercepted are imported into the computer, and then, the neural network algorithm in machine language is used to recognize, extract, and synthesize the trained sequence voice audio and text frame video. Comparing the data in Table 3, it is found that the accuracy of the improved algorithm in intercepting effective speech signals has been improved, which lays a foundation for the subsequent improvement of system performance.

**TABLE 3:** Voice signal interception accuracy.

Noise circumstance	Energy (%)	SNR/dB	Inverted spectrum distance (%)	The algorithm in this paper (%)
Outdoor environment	67.2	5	64.2	87.4
	84.2	10	65.3	90.1
	74.3	0	70.5	81.3
	87.3	5	75.2	85.4
Indoor environment	84.2	0	81.5	89.1
	78.9	5	87.2	87.1
	92.2	0	75.6	92.5
	91.0	10	84.6	93.1

### User Experience Analysis Based on Artificial Intelligence Voice Interaction

In the process of designing speech intelligent recognition system, we should first consider whether the design terminal has artificial intelligence thinking and then consider the impact on user functions. Therefore, this paper mainly focuses on the investigation and analysis of customers’ perception and experience on the financial self-service terminal. Through the investigation of the function and use of intelligent voice interaction, users will feel in varying degrees after accepting the artificial intelligence design, mainly in terms of feeling, operation convenience, and comfort.

User experience refers to people’s understanding of things, which includes their cognition of itself and the surrounding environment, and also their emotional satisfaction through the use of these products or services. Questionnaire Table 4 shows that male users in the electronics industry or partial science industry believe that most of the current interaction methods are keyboard or gesture voice. At the same time, the understanding of female users in liberal arts industries is expression interaction, eye tracking, or action interaction and tablet. From the results of the questionnaire survey, we can try to draw the following conclusions. For the current intelligent voice system, we pay more attention to its function rather than designing the feeling experience. At the same time, we also have high requirements for perception, intelligence, and stability. Therefore, in addition to the financial field, the impact of user interaction based on artificial intelligence technology is mainly considered in this survey.

**TABLE 4:** The AI voice interaction questionnaire survey.

Question	Option			
Gender	Male		Female	
Age	20–30 years old	30–40 years old	20–30 years old	30–40 years old
Occupation	Student	White collar	Student	White collar
Profession	Software, electronic communication, automation, mechanical and electrical categories	Medical care, food, and the environment	Finance, literature and history, secretary, text editor	Painter, literature and history teacher
Understanding how you interact	Keyboard, mouse	Voice, gesture	Appearance, eye tracking	Action, handwritten board
Voice interaction application value	There are visual damage and defects, novel interactive ways to benefit game applications	When the eyes are occupied by other things, they need to react flexibly	In some cases, it is not convenient to use keyboard and mouse	More extensive application value

**FIGURE 6:**
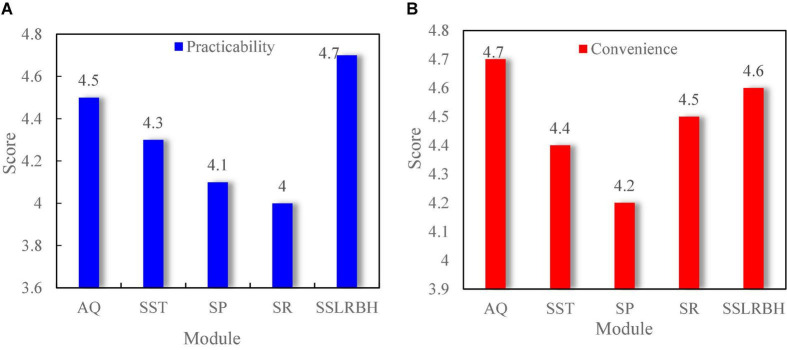
Integration score of financial self-service terminal. **(A)** Practicability. **(B)** Convenience.

The self-service terminal takes “24-h self-service” as the system design concept, which can alleviate the problem of excessive flow of people in the traditional business hall, overcome the shortcomings of the original business hours, greatly reduce the difficulties of customers when they do business in the business hall, and enable customers to enjoy more simple, convenient, and considerate services. In financial business, users can also realize accounting query, automatic transfer, statement printing, supplementary registration, and automatic loss reporting. Through equipment value-added development, it can also use supporting facilities for product procurement and other value-added services. The equipment also has the advantages of saving staff expenses, which reduces operating costs, 24-h continuous work, error-free operation, and so on. As can be seen from Figure 6, the practicability (Figure 6A) and convenience (Figure 6B) of each module of the financial self-service terminal integration in the user experience survey score more than four points, which shows that users have a good sense of experience of the self-service terminal.

**FIGURE 7:**
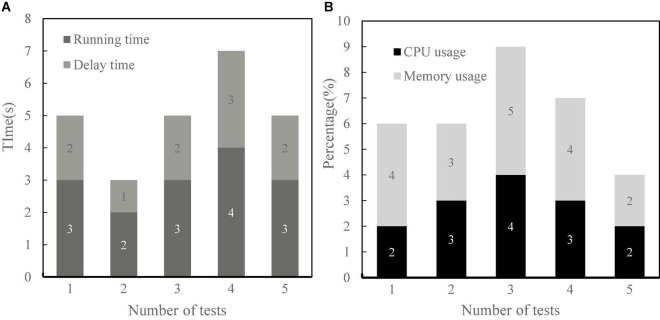
The flow of people and the degree of financial self-service terminals of artificial intelligence. **(A)** Visitors flow-rate. **(B)** Degree of artificial intelligence.

Intelligent self-service terminal is a virtual system. Its functions include voice recognition, fingerprint acquisition, and automatic password. Generally, the terminal is placed in public places with large traffic such as telecom business hall, toll collection point, station, airport, and large shopping mall, so as to facilitate people’s trading activities. As can be seen from Figure 7, the financial self-service terminals in airports and large shopping malls have the largest number of users and large traffic. Correspondingly, the terminal AI interaction frequency in these two public places is also high. This shows that places with large flow of people have a high degree of artificial intelligence.

### Integrated Performance Analysis of Financial Self-Service Terminal

**FIGURE 8:**
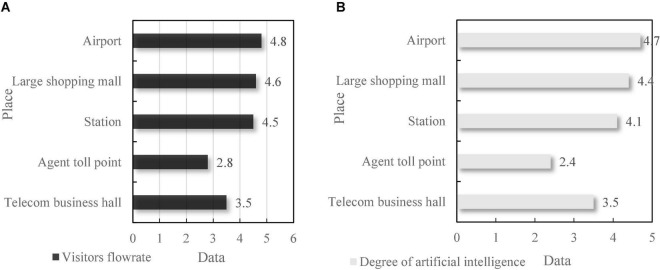
Integrated performance analysis of financial self-service terminals. **(A)** Time. **(B)** Occupancy rate.

The function of financial self-service terminal is mainly to authenticate customers and realize interaction through voice communication, so the differences between different users must be fully considered in the design. This paper not only tests the voice technology and user experience, but also tests the performance of the terminal from the running time, delay time, memory occupation rate, and processing unit (CPU) occupation rate of the terminal. As can be seen from Figure 8, the terminal has good performance in all aspects, short running time and delay time, and small memory, and CPU occupation. This shows that the terminal can meet the needs of users.

**FIGURE 9:**
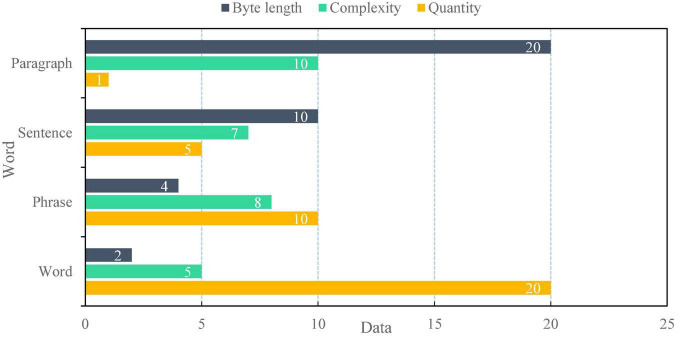
Voice materials.

**FIGURE 10:**
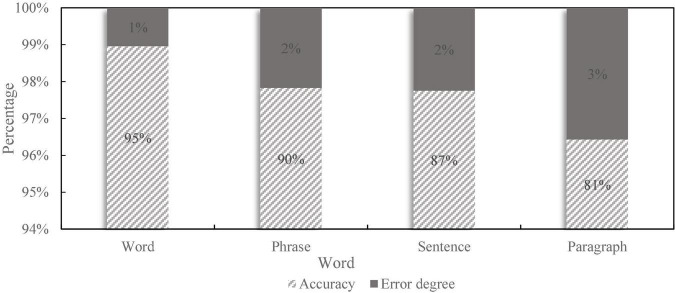
Accuracy and error rate contrast.

To test the actual voice application effect of the financial self-service terminal based on artificial intelligence voice interaction, after the terminal design, 20 words, 10 groups of words, five sentences, and one paragraph as shown in Figure 9 are selected, respectively. The complexity is based on the corresponding words and sentence difficulty. Then, the speech accuracy and error rate of the above materials are detected, and the detection results are shown in Figure 10.

In the process of speech recognition, due to certain errors, the recognition results will be biased, which seriously affects the performance of the system. For a low error rate algorithm, the accuracy rate can be used to indicate whether the computer has correct machine codeword or text content integrity. When the code has been modified or there are other problems, it is necessary to reuse the speech recognition method to correct these differences. Therefore, the influence of various factors on the results should be fully considered in the identification process. As can be seen from Figure 10, the accuracy of the speech recognition function of the financial self-service terminal basically reaches more than 80%, and the accuracy decreases with the increase of the difficulty of the corpus, but it can basically meet the daily needs.

## Discussion

With the development and progress of science and technology, all kinds of robots have gradually appeared in our life, and the concept of “human–computer interaction” has gradually penetrated into our production and life. How to connect the real physical world and virtual information world more closely becomes one of the focuses in the field of science. Human–computer interaction refers to the two-way communication between the computer and the user, that is, the two-way information exchange, that is, the user can input information to the computer, or the computer can feed back information to the user. The development of artificial intelligence technology has brought convenience to people’s life, but with its higher popularity and richer application scenarios. In the future, scientific and technological products and services will become more intelligent than human. The integrated design of artificial intelligence financial self-service terminal is a very promising field. It can make it more convenient for customers to use the system and also allow users to update and interact with the interface in real time. Small volume and multifunctional embedded equipment becomes an inevitable trend in the market, which makes the application of voice interaction technology based on embedded platform widely expanded. Speech interaction technology mainly includes speech interaction equipment, speech recognition, speech synthesis, speaker recognition, and other related technologies. Because the previous speech recognition technology and language fusion technology are implemented locally, the requirements for the microprocessor on the local port are quite high. Therefore, the CPU needs to have strong data processing capacity and sufficient storage capacity to store the vocabulary as large as possible. At the same time, due to the increasing technical level of language acquisition module, and also the latest language recognition technology and fusion software, language terminals need to constantly improve their software and hardware, which seriously limits the development of language technology application. With deep learning and cloud computing technology, the effective application of the core effect of language interaction technology in language interaction has made great progress. Integrating language interaction technology into the current hot cloud computing technology will create a new model for language service and make human–computer language interaction technology which has a bright future and huge market prospect in the fast and developed network information age.

As an interactive mode, speech recognition plays an important role. Speech processing technology is an important research direction in natural language processing technology. In short, speech recognition engineering is an interdisciplinary comprehensive engineering technology, which includes sound feature extraction, acoustic modeling, language simulation and language coding, and other knowledge systems. As one of the most natural human–computer interaction methods, language has developed rapidly under the promotion of artificial intelligence technology in recent years. At present, language interaction technology is imperceptibly changing human living habits from various fields. Language is the main technical means to convey information to people, and speech recognition and language fusion are also the core technologies of human language control. The human–computer interaction of natural speech is also a hot topic in the current research.

## Conclusion

With the rapid development and popularization of artificial intelligence technology, artificial intelligence will be an indispensable and important part of human social life in the future. Intelligent will be widely used in people’s daily life and work, so it is very important to make full use of computers, AI, and other scientific and technological tools. Based on the function and design of the existing intelligent terminal, combined with the development trend of the current financial industry, this paper puts forward an integrated design of voice interaction technology based on artificial intelligence. With the continuous progress and innovation of science and technology in the future, it is necessary to understand the latest needs and product concepts deeply in this field to take into social integration perfectly.

## Data Availability Statement

The original contributions presented in the study are included in the article/supplementary material, further inquiries can be directed to the corresponding author.

## Author Contributions

All authors listed have made a substantial, direct, and intellectual contribution to the work, and approved it for publication.

## Conflict of Interest

The authors declare that the research was conducted in the absence of any commercial or financial relationships that could be construed as a potential conflict of interest.

## Publisher’s Note

All claims expressed in this article are solely those of the authors and do not necessarily represent those of their affiliated organizations, or those of the publisher, the editors and the reviewers. Any product that may be evaluated in this article, or claim that may be made by its manufacturer, is not guaranteed or endorsed by the publisher.
